# The Combination of Bioavailable Concentrations of Curcumin and Resveratrol Shapes Immune Responses While Retaining the Ability to Reduce Cancer Cell Survival

**DOI:** 10.3390/ijms25010232

**Published:** 2023-12-23

**Authors:** Chiara Focaccetti, Camilla Palumbo, Monica Benvenuto, Raffaele Carrano, Ombretta Melaiu, Daniela Nardozi, Valentina Angiolini, Valeria Lucarini, Bora Kërpi, Laura Masuelli, Loredana Cifaldi, Roberto Bei

**Affiliations:** 1 Department of Clinical Sciences and Translational Medicine, University of Rome “Tor Vergata”, 00133 Rome, Italy; chiara.focaccetti@uniroma2.it (C.F.); camilla.palumbo@uniroma2.it (C.P.); monica.benvenuto@unicamillus.org (M.B.); raffo9318@gmail.com (R.C.); ombretta.melaiu@uniroma2.it (O.M.); daniela.nardozi@uniroma2.it (D.N.); cifaldi@med.uniroma2.it (L.C.); 2 Departmental Faculty of Medicine and Surgery, Saint Camillus International University of Health and Medical Sciences, 00131 Rome, Italy; 3 Department of Experimental Medicine, University of Rome “Sapienza”, 00161 Rome, Italy; valentina.angiolini@uniroma1.it (V.A.); valeria.lucarini@uniroma1.it (V.L.); laura.masuelli@uniroma1.it (L.M.); 4 Department of Biomedicine, Catholic University ‘Our Lady of Good Counsel’, 1000 Tirana, Albania; b.kerpi@unizkm.al; 5 Faculty of Medicine and Surgery, Catholic University ‘Our Lady of Good Counsel’, 1000 Tirana, Albania

**Keywords:** polyphenols, Curcumin, Resveratrol, PBMCs, immune response, T cells, NK cells

## Abstract

The polyphenols Curcumin (CUR) and Resveratrol (RES) are widely described for their antitumoral effects. However, their low bioavailability is a drawback for their use in therapy. The aim of this study was to explore whether CUR and RES, used at a bioavailable concentration, could modulate immune responses while retaining antitumor activity and to determine whether CUR and RES effects on the immune responses of peripheral blood mononuclear cells (PBMCs) and tumor growth inhibition could be improved by their combination. We demonstrate that the low-dose combination of CUR and RES reduced the survival of cancer cell lines but had no effect on the viability of PBMCs. Although following CUR + RES treatment T lymphocytes showed an enhanced activated state, RES counteracted the increased IFN-γ expression induced by CUR in T cells and the polyphenol combination increased IL-10 production by T regulatory cells. On the other hand, the combined treatment enhanced NK cell activity through the up- and downregulation of activating and inhibitory receptors and increased CD68 expression levels on monocytes/macrophages. Overall, our results indicate that the combination of CUR and RES at low doses differentially shapes immune cells while retaining antitumor activity, support the use of this polyphenol combinations in anticancer therapy and suggest its possible application as adjuvant for NK cell-based immunotherapies.

## 1. Introduction

Polyphenols are a considerable group of natural compounds found in foods and beverages of vegetal origin. Numerous studies have shown that polyphenols have potent antioxidant, anti-inflammatory, antimicrobial and anticancer properties [1], so their consumption is considered beneficial for the human body [2].

Curcumin (CUR) (l,7-bis-(4-hydroxy-3-methoxyphenyl)-l,6-heptadiene-3,5-dione) is a yellow polyphenol found in turmeric, a spice purified from the rhizome of the plant Curcuma longa of the Zingiberaceae family. CUR is a pleiotropic molecule, able to target several signaling pathways involved in carcinogenesis [3,4,5,6]. Indeed, CUR is able to suppress proliferation, to induce apoptosis, to inhibit epithelial mesenchymal transition (EMT), neoangiogenesis, invasion and metastasis in different types of cancer [7,8,9,10,11,12]. The stilbene Resveratrol (RES) (3,5,4′-trihydroxy-trans-stilbene) is found in grapes, berries, peanuts, plums and pine nuts, as cis, trans isomers or conjugated derivatives [3,5]. Like CUR, RES has valuable biological properties, being able to counteract cancer development and progression by affecting several signaling pathways [3,6,13].

However, the beneficial effects of polyphenols are limited by their poor bioavailability. Indeed, polyphenols have a poor biodistribution and absorption, as well as a quick metabolism and elimination in the human body. The mechanisms that limit the bioavailability of oral administered polyphenols encompass their metabolism in the gastrointestinal tract and liver, their binding to blood cell surfaces, the action of the microbial flora in the mouth and gut, and additional regulatory factors that reduce the toxicity of high doses of compounds on mitochondria or other organelles [14]. Further, in addition to endogenous factors, dietary variables, such as food matrix and food preparation methods, might also alter the bioavailability of polyphenols [4,14]. Thus, after dietary intake, only nano- or micromolar quantities of polyphenols and their metabolites are detected in plasma [15]. In this regard, as reported by several reviews, the results of the investigations in humans differ significantly, with a Cmax in plasma ranging from 1.17 µM to 5.6 µM (oral intake of 2–5 g) for RES [16] and from 2.7 nM to 8.7 µM (oral intake of 2–10 g) for CUR [17]. Although these discrepancies could be attributable to different quantification approaches, their actual causes are not known [16,17].

Since the low bioavailability of polyphenols negatively impacts on the effective dose delivered to cancer cells and it is regarded as one of the main factors able to limit their effectiveness in cancer patients [4], several attempts have been made to develop formulations, derivatives and analogues with enhanced bioavailability, solubility and stability [3,5,18,19,20]. Another strategy for enhancing polyphenols effects on cancer cells is their use in combination, since different polyphenols combined together at low doses might have a synergic or additive effect [21,22,23,24]. In fact, several studies demonstrated that treatment with polyphenol combinations is more effective in suppressing cancer growth than treatment with a single polyphenol compound [3,25,26]. For instance, the combination of CUR and RES had more potent cytotoxic effects than either compound alone on hepatocellular carcinoma [21] and colorectal cancer cells [22] and was reported to be able to synergistically restrain cervical cancer cells proliferation and migration [23,24]. CUR plus RES treatment was also demonstrated more effective than the single drugs in reducing the proliferation of colon cancer cells in vitro and in vivo [22]. Combination treatments also suppressed chemoresistance to cisplatin of ovarian cancer cells [27]. In this context, our group previously demonstrated that the combination of diallyl disulfide (DADS) plus RES, DADS plus CUR, and RES plus CUR displayed stronger in vitro anticancer activity on malignant rhabdoid or osteosarcoma cell lines than the single polyphenols and that RES and DADS increased the apoptotic effects of CUR [28]. We also reported that RES enhanced CUR anticancer activities on head and neck cancer in vitro and in vivo. Moreover, RES plus CUR therapy inhibited the development of transplanted salivary gland cancer cells in mice more effectively than either CUR or RES alone [29]. Furthermore, we showed that CUR with RES affected the PI3K/AKT/mTOR pathway, autophagy, intracellular reactive oxygen species (ROS) and ER stress/UPR both in breast and salivary gland tumor cell lines derived from Her-2/neu transgenic mice and that RES increased CUR cytotoxic effect by suppressing CUR-induced pro-survival autophagy [30]. Still, it should be emphasized that in most of the in vitro studies aimed at evaluating the anticancer effects of these compounds, the polyphenols are used at concentrations higher than those attainable in vivo [6].

Recently, several studies have shown that polyphenols, including CUR and RES, also have the ability to modulate immune responses and may enhance antitumor immunity while preventing or delaying the development of tumor-supporting leukocytes by influencing the activity of immune cells, the production of cytokines, and the regulation of other elements of the immunological defense system [8,10,31,32,33,34,35,36,37,38,39,40,41,42,43,44,45,46,47,48,49,50,51,52,53,54,55,56,57,58,59,60,61,62,63,64,65,66,67,68,69,70,71]. Indeed, the tumor immune microenvironment is composed of different immune cells, which can play a dual role in the development of cancer. Anticancer cells, such as Natural Killer (NK) cells and CD8^+^ T lymphocytes, can recognize and eliminate tumor cells; on the other side, immunosuppressive cells, such as regulatory T cells (Tregs), myeloid-derived suppressor cells (MDSCs) and tumor-associated macrophages (TAMs), can support the evasion of immune surveillance by neoplastic cells and promote tumor growth [2].

Given these evidences, the aim of our study was (a) to explore whether CUR and RES, used at their bioavailable concentration (5 µM) [16,17] could modulate immune responses while retaining antitumor activity and (b) to determine whether CUR and RES effects on the immune responses of peripheral blood mononuclear cells (PBMCs) and tumor growth inhibition could be improved by their low-dose combination.

## 2. Results

### 2.1. Effect of Low-Dose CUR and RES on Tumor Cell Survival

**Figure 1:**
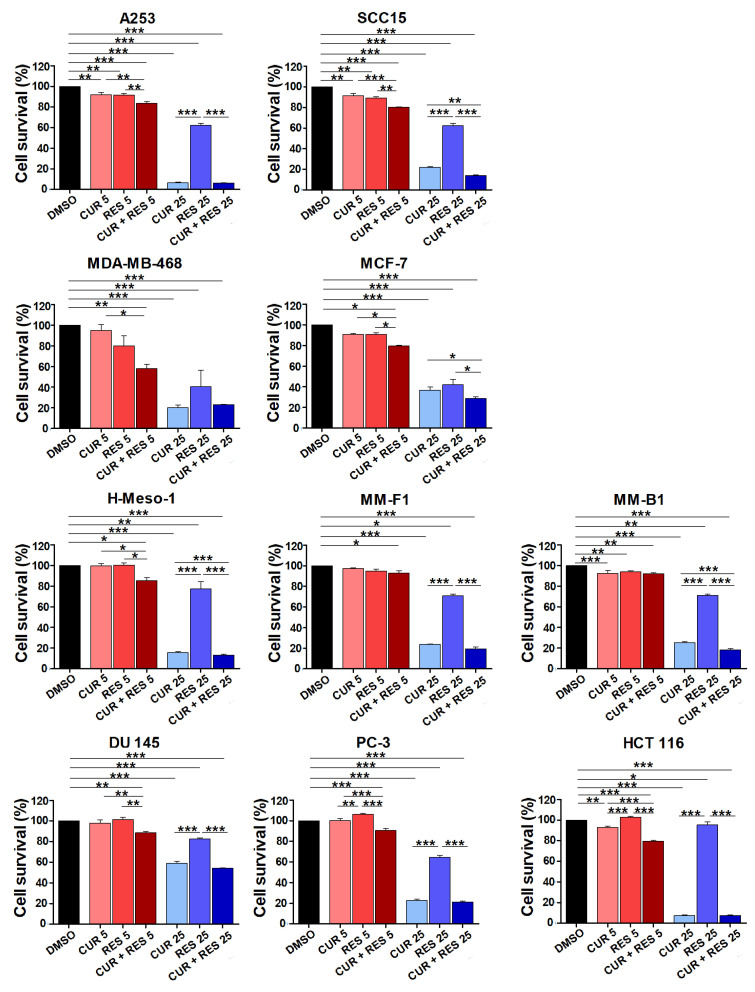
Effect of low-dose CUR and RES on tumor cell survival. Cell survival was evaluated by the SRB assay on a panel of tumor cell lines including the head and neck carcinoma (SCC-15, A253), breast cancer (MCF-7, MDA-MB-468), malignant mesothelioma (MM-B1, MM-F1, H-Meso-1), prostate cancer (DU 145, PC-3) and colon cancer (HCT 116) cell lines, after 96 h of treatment with DMSO or CUR and/or RES at 5 and 25 µM. The percentage survival of polyphenol-treated cells was calculated relative to that of DMSO-treated control cells. Results are expressed as the mean ± SD of three independent experiments performed in triplicate. Statistical significance was calculated with one-way ANOVA (* *p* ≤ 0.05, ** *p* ≤ 0.01, *** *p* ≤ 0.001). The effect of CUR, RES, and CUR + RES 25 µM vs. DMSO was always significant.

The effects of CUR and RES on tumor cell growth were evaluated at the 5 µM bioavailable concentration and, for comparison, at a 5-fold higher concentration using a panel of ten human cell lines including head and neck carcinoma (SCC-15, A253), breast cancer (MCF-7, MDA-MB-468), malignant mesothelioma (MM-B1, MM-F1, H-Meso-1), prostate cancer (PC-3, DU 145) and colon cancer (HCT 116) cell lines. Cell survival was assessed by the SRB assay after 96 h of treatment with the polyphenols, alone or combined in equimolar concentrations, or with DMSO used as solvent of the compounds. In all tumor cell lines both compounds, either alone or in combination, were able to significantly reduce cell survival when used at the high dose (25 µM) (Figure 1). At 25 µM, CUR was more effective than RES on cell survival inhibition. The effect obtained with 25 µM CUR + RES was significantly higher than the effect of CUR in SCC-15, MCF-7, M-Meso-1 and MM-B1cells.

As for the effects of the low dose of CUR and RES (Figure 1), when the two compounds were used alone at 5 µM, a modest but significant cell survival inhibition was observed in only 3 out of the 10 cell lines tested, i.e., the head and neck carcinoma cell lines A253 and SCC15 and the mesothelioma cell line MM-B1. Still, when CUR and RES were combined at the 5 µM bioavailable concentration, their inhibitory effect was significant on all cell lines. Furthermore, on seven cell lines (A253, SCC15, MCF-7, H-Meso-1, DU 145, PC-3, and HCT 116) the low-dose combination of CUR and RES was more potent than either compound used alone. Even though, on the whole, the percentage reduction in tumor cell survival obtained with CUR + RES at the low dose was modest, the reported findings suggest that long-term supplementation with this combination of polyphenols may have a clinical impact in cancer patients.

### 2.2. Effects of Low-Dose CUR and RES on Proliferation and Death of PBMCs

**Figure 2:**
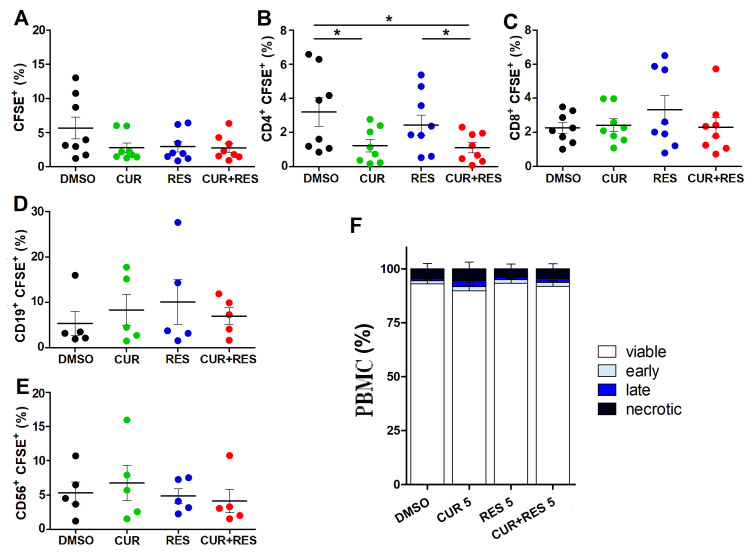
Effect of low-dose CUR and RES on PBMC proliferation and cell death. (**A**–**E**) Cell proliferation of resting PBMCs was assessed by flow cytometry using the dilution of CFSE dye after 96 h of treatment with DMSO, CUR and/or RES (5 µM). The results are presented as the mean ± SD of the frequency of cells subsets in PBMCs from five or eight healthy donors. (**A**) Total lymphocytes identified based on morphological characteristics on FSC/SSC; (**B**) CD3^+^CD19^−^CD14^−^CD4^+^ helper T lymphocytes, (**C**) CD3^+^CD19^−^CD14^−^CD8^+^ cytotoxic T lymphocytes, (**D**) CD3^−^CD14^−^CD19^+^ B cells and (**E**) CD3^−^CD19^−^CD14^−^CD56^+^ NK cells, identified by positive staining for the respective markers. Statistical significance of the effects obtained with CUR and RES, alone or in combination, was calculated with two-tailed unpaired Student’s *t* test (* *p* ≤ 0.05). (**F**) Percentages of viable, necrotic, early and late apoptotic cells after 96 h of treatment with DMSO, CUR and/or RES (5 µM) as assessed by the Annexin V/AAD assay and flow cytometry. Results are expressed as the mean ± SD of the independent analysis of PBMCs from eight healthy donors. Statistical significance of the effects obtained with CUR and RES, alone or in combination, was calculated with one-way ANOVA.

The effects of low-dose CUR and RES on PBMC proliferation were next evaluated. Resting PBMCs were treated with the polyphenols at 5 µM for 96 h. Flow cytometric measurement of CFSE dye dilution was then used to assess cell proliferation of the total lymphocyte population as well as that of helper T lymphocytes (CD3^+^CD19^−^CD14^−^CD4^+^), cytotoxic T lymphocytes (CD3^+^CD19^−^CD14^−^CD8^+^), B lymphocytes (CD3^−^CD14^−^CD19^+^) and NK cells (CD3^−^CD19^−^CD14^−^CD56^+^) subpopulations (Supplementary Figure S1). CUR, alone (1.2 ± 1.0%) or combined with RES (1.1 ± 0.9%) reduced the percentage of proliferating CD4^+^ T cells, as compared to DMSO (3.2 ± 2.4%) and RES alone (2.7 ± 1.7%), without significantly affecting CD8^+^ T, B and NK cells (Figure 2A–E).

In PBMCs treated with 5 µM CUR and RES, alone or in combination, a very low level of cell death was detected, which was not significantly different from that of the DMSO-treated controls (Figure 2F). According to these findings, PBMCs survival is not affected by the low, bioavailable concentrations of the two polyphenols. Interestingly, the low-dose combination of CUR and RES was able to decrease oxidative stress in PBMCs, whereas the single compounds had no significant effects in this regard (Supplementary Figure S2).

### 2.3. Effects of Low-Dose CUR and RES on Activation and Functional Properties of Resting T Lymphocytes

**Figure 3:**
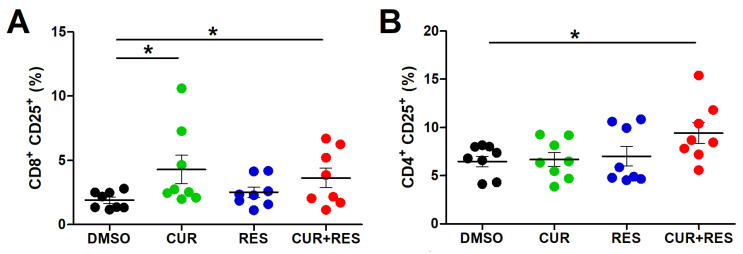
Effect of CUR and RES on CD25 activation marker expression in T lymphocytes. CD25 expression in resting (**A**) CD3^+^CD19^−^CD14^−^CD8^+^ and (**B**) CD3^+^CD19^−^CD14^−^CD4^+^ T lymphocytes was assessed by flow cytometry after 96 h of PBMCs treatment with DMSO, CUR, and/or RES (5 µM). The results are presented as the mean ± SD of the frequency of cells subsets in PBMCs from eight healthy donors. Statistical significance of the effects obtained with CUR and RES, alone or in combination, was calculated with two-tailed unpaired Student’s *t* test (* *p* ≤ 0.05).

The CD25 receptor, also known as Interleukin-2 receptor (IL-2R), is not expressed by quiescent mature T lymphocytes, but its expression is rapidly induced upon cell activation [72]. Thus, the percentage of lymphocytes expressing CD25 provides an indication of their activation status. Hence, the modulation of CD25 expression was evaluated in resting lymphocytes from healthy donors after 96 h of treatment with 5 µM CUR and RES, alone or in combination. CUR (4.3 ± 3.1%) and CUR + RES (3.6 ± 2.2%) significantly increased the percentage of CD3^+^CD19^−^CD14^−^CD8^+^ T lymphocytes expressing the activation marker as compared to DMSO (1.9 ± 0.7%) (Figure 3A). Moreover, the percentage of CD3^+^CD19^−^CD14^−^CD4^+^ T helper cells positive for CD25 was not significantly modified by either compound used alone, while it was significantly increased after treatment with the CUR + RES combination (9.4 ± 3.1%) as compared to DMSO (6.8 ± 1.5%) (Figure 3B).

**Figure 4:**
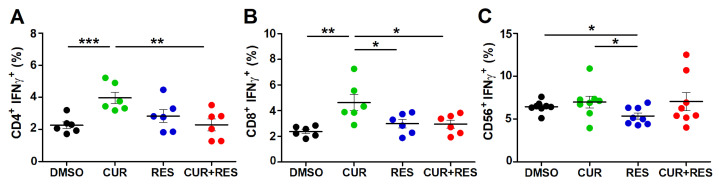
Effect of CUR and RES on IFN-γ expression in T lymphocytes and NK cells. IFN-γ expression was assessed by flow cytometry after 96 h of PBMCs treatment with DMSO, CUR and/or RES (5 µM) on (**A**) resting CD3^+^CD19^−^CD14^−^CD4^+^ T lymphocytes, (**B**) resting CD3^+^CD19^−^CD14^−^CD8^+^ T lymphocytes, and (**C**) NK cells. The results are presented as the mean ± SD of the frequency of IFN-γ^+^ cell subsets in PBMCs obtained from six to eight healthy donors. Statistical significance of the effects obtained with CUR and RES, alone or in combination, was calculated with two-tailed unpaired Student’s *t* test (* *p* ≤ 0.05; ** *p* ≤ 0.01; *** *p* ≤ 0.001).

The functional status of T lymphocytes and NK cells is also assessed in terms of production of the anticancer cytokine IFN-γ. After 96 h of PBMCs treatment with the compounds at 5 µM, CUR significantly increased the percentage of IFN-γ-producing CD3^+^CD19^−^CD14^−^CD4^+^ (4.0 ± 0.9%) and CD3^+^CD19^−^CD14^−^CD8^+^ (4.6 ± 1.6%) T lymphocytes compared to the control (DMSO-CD4^+^: 2.3 ± 0.5%; DMSO-CD8^+^: 2.4 ± 0.4%) (Figure 4A,B). Conversely, IFN-γ production was not modified by RES and CUR + RES treatments in both CD4^+^ and CD8^+^ T cells (Figure 4A,B). This observation suggests a neutralizing effect of RES on CUR-mediated induction of IFN-γ expression. Conversely, in NK cells neither CUR nor CUR + RES treatments affected the production of IFN-γ, while RES (5.4 ± 1.0%) significantly reduced the cytokine expression in comparison to both DMSO (6.4 ± 0.7%) and CUR (7.0 ± 1.9%) (Figure 4C). This observation suggests that CUR can counteract the RES-mediated reduction in IFN-γ expression.

### 2.4. Effect of Low-Dose CUR and RES on Frequency and Functional Properties of Regulatory T Cells

**Figure 5:**
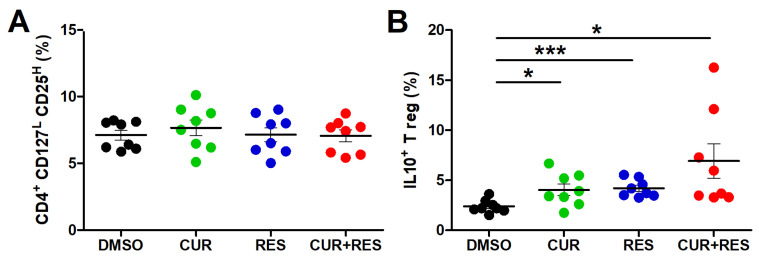
Effect of CUR and RES on Tregs frequency and IL-10 production. PBMCs were treated for 96 h with DMSO, CUR, and/or RES (5 µM) and analyzed by flow cytometry to assess (**A**) the frequency of CD4^+^CD25^high^CD127^low/neg^ cells; (**B**) the frequency of IL-10^+^ Tregs. The results are presented as the mean ± SD of the frequency of cells in PBMCs from eight healthy donors. Statistical significance of the effects obtained with CUR and RES, alone or in combination, was calculated with two-tailed unpaired Student’s *t* test (* *p* ≤ 0.05; *** *p* ≤ 0.001).

Regulatory T cells (Tregs) have a crucial role in peripheral immune tolerance and mediate the establishment of an immunosuppressive microenvironment that favors tumor immune escape [73]. The treatment of PBMCs with CUR and RES, alone or in combination at 5 µM for 96 h, did not affect the frequency of Tregs, identified by the combined expression of the markers CD4^+^CD25^high^CD127^low/neg^ (Figure 5A). However, both the single and combined treatments increased the expression of the immunosuppressive cytokine IL-10 by Tregs, the highest increase being induced by the combined treatment. In fact, the frequency of IL-10-positive Tregs in PBMCs treated with CUR + RES was approximately 3-fold higher than that observed in PMBCs treated with DMSO only (CUR + RES vs. DMSO: 6.9 ± 4.8% vs. 2.4 ± 0.7%) (Figure 5B).

### 2.5. Effect of Low-Dose CUR and RES on NK Cell-Mediated Recognition of Tumor Target Cells

**Figure 6:**
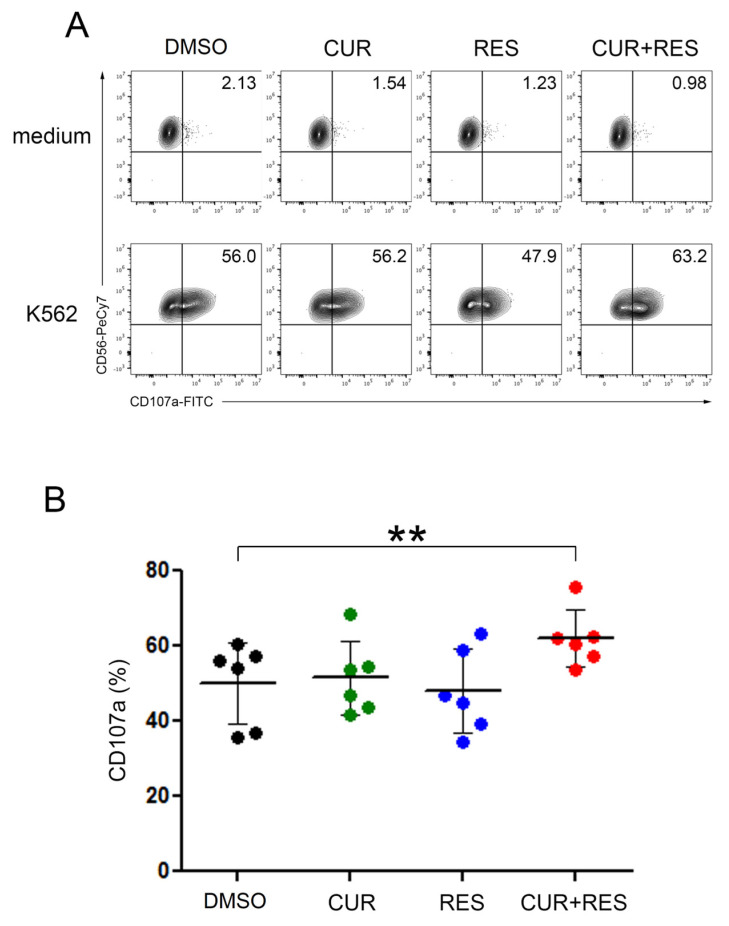
Enhanced degranulation of NK cells upon CUR and RES treatment. (**A**) PBMCs, pre-treated with 5 µM CUR and/or RES for 48 h, were evaluated for NK cell-mediated degranulation assay against K562 cells or medium alone as control. The percentage of CD107a in the NK cell subset is indicated in each plot. A representative experiment out of six performed with PBMCs isolated from six healthy donors is shown. (**B**) Summary of degranulation studies of NK cells from PBMCs isolated from six healthy donors. Dots correspond to the percentage of CD107a^+^ NK cells in PBMCs from each donor, and the mean ± SD are also reported. Statistical significance of the effects obtained with CUR and RES, alone or in combination, was calculated vs. those obtained with DMSO-treated cells by two-tailed unpaired Student’s *t* test (** *p* < 0.01).

To further assess the immunomodulatory effect of CUR and RES on NK cells, human PBMCs treated for 48 h with 5 µM CUR and RES, alone or in combination, were used in degranulation assays against K562 target cells and stained to specifically assess the functional contribution of the NK cell subset (the gating strategy is shown in Supplementary Figure S3). As evaluated by the percentage of cells positive for the degranulation marker CD107a, NK cells treated with the CUR + RES combination were significantly more activated than control cells, while no significant differences were observed between DMSO-treated NK cells and NK cells treated with either CUR or RES alone (Figure 6).

**Figure 7:**
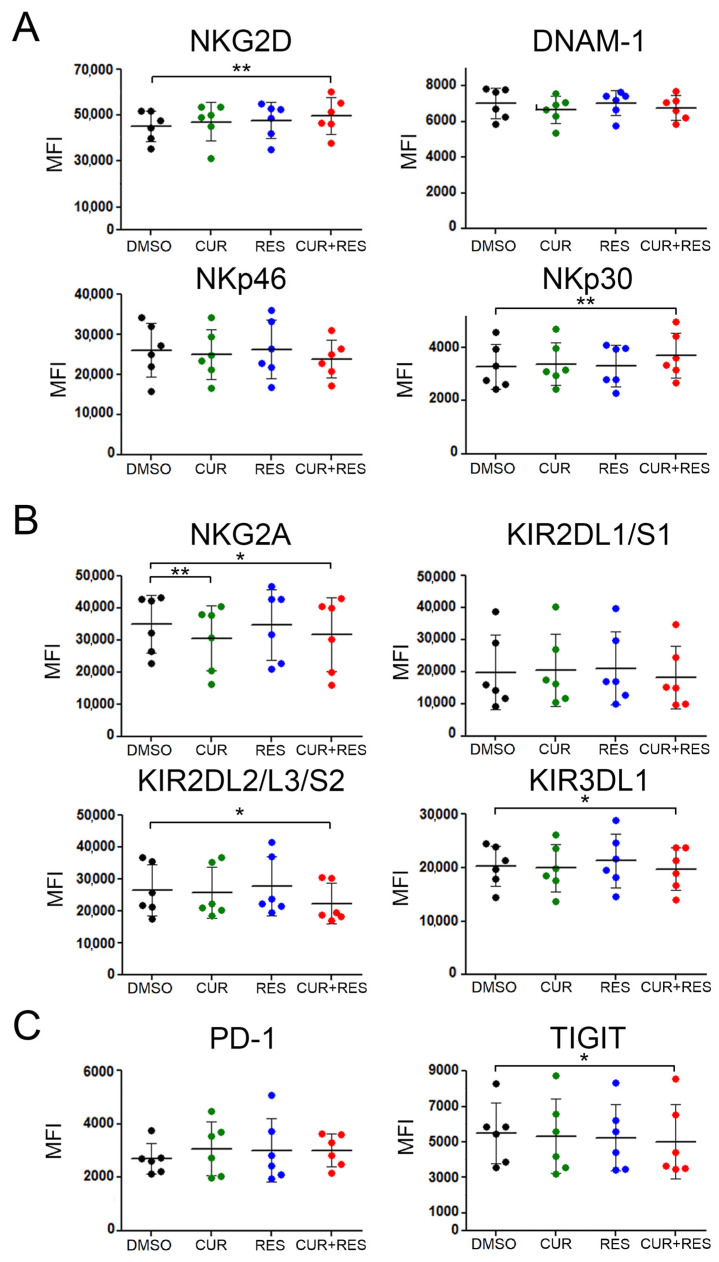
Surface expression of NK cell receptors upon CUR and RES treatment. PBMCs, pre-treated with CUR and/or RES at 5 µM for 48 h, were stained for NK cell activating receptors such as (**A**) NKG2D, DNAM-1, NKp46 and NKp30, (**B**) NK cell inhibitory receptors such as NKG2A and KIRs, and (**C**) NK cell exhaustion receptors such as PD-1 and TIGIT. Dots correspond to the mean of fluorescence (MFI) of the indicated receptors expressed on NK cells in PBMCs isolated from six healthy donors. Data are expressed as the mean ± SD bars. Statistical significance of the effects obtained with CUR and RES, alone or in combination, was calculated vs. those obtained in DMSO-treated cells with two-tailed unpaired Student’s *t* test (* *p* < 0.05, ** *p* < 0.01).

Then, we evaluated whether the treatment with CUR and RES could affect the expression of molecules involved in the recognition of tumor target cells by NK cells, including activating receptors (NKG2D, DNAM-1, NKp30 and NKp46), inhibitory receptors (NKG2A, KIRs) and exhaustion receptors (PD-1 and TIGIT) [74]. The combined CUR + RES treatment significantly increased the expression of activating receptors such as NKG2D and NKp30 (Figure 7A). Conversely, the expression of inhibitory receptors such as NKG2A, KIR2DL2/L3/S2 and KIR3DL1 as well as the expression of exhaustion receptors such as TIGIT were significantly reduced (Figure 7B,C). Of note, CUR treatment alone induced a significant reduction in NKG2A expression, consistent with previously reported results [31]. These findings indicate that the combined low-dose CUR + RES treatment significantly enhanced NK cell activation through the upmodulation of activating receptors and concomitant reduction of inhibitory and exhaustion receptors.

### 2.6. Effect of Low-Dose CUR and RES on Monocytes/Macrophages

**Figure 8:**
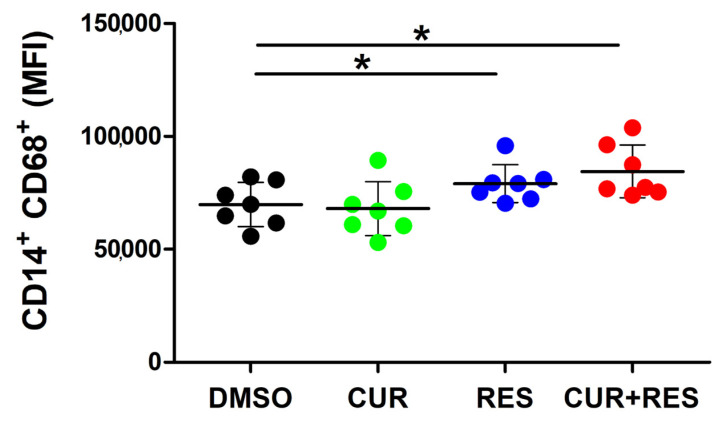
Effect of CUR and RES on expression of CD68 in monocyte/macrophage cells. PBMCs, pre-treated for 48 h with DMSO, CUR, and/or RES (5 µM), were analyzed by flow cytometry to assess the expression of CD68 on monocytes/macrophages (CD3^−^CD19^−^CD56^−^CD14^+^). Dots correspond to the MFI of CD68 expressed on the monocyte/macrophage subset in PBMCs isolated from seven healthy donors. Data are presented as the mean ± SD bars. Statistical significance of the effects obtained with CUR and RES, alone or in combination, was calculated vs. those obtained in DMSO-treated cells with two-tailed unpaired Student’s *t* test (* *p* < 0.05).

To further explore the effect of the polyphenols at low doses on innate immune cells, human PBMCs were treated for 48 h with 5 µM CUR and RES, alone or in combination, and then stained to evaluate the expression of CD68, a glycosylated type I transmenbrane glycoprotein associated with the endosomal/lysosomal compartment in the monocyte/macrophage subset [75]. Both RES and CUR + RES induced a significant increase of CD68 expression levels in CD3^-^CD56^-^CD19^-^CD14^+^ monocytes/macrophages (Figure 8).

## 3. Discussion

Polyphenols are a large group of compounds and secondary plant metabolites responsible for the color and flavor of fruits, flowers, and vegetables [1,2]. They also play roles in plant defense against pathogens, possess antioxidant properties and modulate multiple signaling processes. Among these molecules are stilbenes, like RES, and curcuminoids, like CUR. Herein, we explored the antitumor efficacy of a combined, bioavailable low-dose treatment with CUR and RES, evaluating their in vitro effects on tumor cell survival as well as on growth, death, and functional properties of lymphocytes from healthy donors’ PBMCs. As compared to the strong reduction in tumor cell survival obtained with high-dose (25 µM) CUR and RES, the two compounds used at a low dose (5 µM) retained a modest efficacy on selected cell lines when used individually but had significantly more consistent effects when used in combination. Remarkably, when the same low-dose treatment conditions were used on PBMCs from healthy donors, CUR, either alone or in combination with RES, reduced the proliferation of CD4^+^ T lymphocytes, but had no significant effects on CD8^+^ T lymphocyte, B lymphocyte and NK cell proliferation. Moreover, the percentage of viable vs. necrotic/apoptotic PBMCs was not affected by the single or combined treatment with the compounds at low doses. While it has been previously reported that CUR and RES do not affect the viability of PBMCs when used individually at concentrations up to approximately 20–25 µM [76,77,78], to our knowledge, this is the first study demonstrating the absence of toxic effects on human PBMCs treated with the two compounds combined at a bioavailable dose. Additionally, the antioxidant properties of RES appeared to be potentiated by its combination with CUR.

In summary, the combination of bioavailable concentrations of CUR and RES retained the ability to reduce cancer cell survival while it had no effects on PBMC viability and negatively affected the proliferation of the CD4^+^ T lymphocyte subset only. Still, a more complex scenario emerged with regard to the impact of the combined treatment on lymphocytes’ functional properties, since the effects of the low-dose combination of CUR and RES in vitro appeared at the same time beneficial and unfavorable if translated into the context of the antitumor immune response. As for the beneficial effects, the combined treatment resulted in an increased frequency of CD4^+^ and CD8^+^ T cells expressing the activation marker CD25. In particular, while the percentage of CD8^+^CD25^+^ T lymphocytes was increased to a similar extent by CUR + RES and by CUR alone, the frequency of CD4^+^CD25^+^ T cells was significantly increased only by the polyphenol combination. Worthy of note, this increase of CD4^+^CD25^+^ T lymphocytes was not associated with an increased frequency of CD4^+^CD25^high^CD127^low/neg^ immunosuppressive Tregs, whose amount was not indeed affected by the compounds, either alone or in combination. On the other hand, as compared to CUR or RES administered singularly, the combined treatment resulted in a greater increase in the fraction of Tregs expressing the immunosuppressive cytokine IL-10. Moreover, RES counteracted the increased IFN-γ expression induced by CUR in both CD4^+^ and CD8^+^ T cells. The increase of IL-10 induced by CUR and RES in vitro could prospectively reflect a potential effect of these polyphenols in vivo, regulating inflammatory processes in autoimmune diseases and tumor associated-inflammation [79,80]. CUR induces IL-10 expression and production in different tissues, thereby modulating several inflammatory pathophysiologic conditions [81,82], while RES, by inducing IL-10 production, exerts a beneficial function on microglia cells in ischemic brain injury [83,84].

The anti-inflammatory properties of RES, in terms of pro-inflammatory cytokine downregulation, have been previously reported. Although in different experimental settings, our results are in agreement with what has been stated previously [49,85,86,87], but to our knowledge, the data reported here represent the first report of an increased IFN-γ production by T cells, after a low-dose CUR treatment of 96 h performed on resting PBMCs.

Interestingly, in NK cells, the combined treatment had a different outcome on IFN-γ expression, since in this innate lymphocyte subset, CUR did not modulate IFN-γ expression when used alone, as previously reported in NK92 human NK cells [88], but it was able to abolish the decreased expression of the cytokine induced by RES. In fact, on the whole, the favorable effects of the polyphenol combination were more consistently observed in NK cells than in adaptive lymphocytes. In fact, unlike the single compounds, the CUR + RES treatment was able to improve NK cell-mediated recognition of tumor target cells, with a concomitant upregulation of the activating receptors NKG2D and NKp30 and downregulation of the inhibitory and exhaustion receptors KIR2DL2/L3/S2, KIR3DL1 and TIGIT. Among the investigated receptors, only the NKG2A inhibitory receptor was downregulated to a similar extent by CUR + RES and CUR alone. Of note, in previously published studies, RES has been shown to exert beneficial effects on NK cells, in terms of cytotoxic activity, modulation of activating receptors expression and cytokines’ release, even when administered alone at low doses [40,48,52,55]. Similarly, the single treatment with low-dose CUR was previously reported to improve NK cell cytotoxic activity [48]. While the discrepancies between these and our findings may be ascribed to different experimental conditions and sensitivity of the assays, our results add to those previously published, indicating that the favorable effects of RES and CUR on NK cells may be potentiated by their low-dose combination. Worthwhile to mention, unlike the low-dose treatments, high concentrations of CUR or RES have been reported to inhibit NK cell functions [40,52,89], further highlighting the importance of investigating polyphenols’ effects in vitro using bioavailable concentrations of the compounds. NK cells are critical components of the innate immune system with a well-established role in tumor surveillance. Indeed, these cells have ability to eliminate tumor cells and have been shown to exert a protecting role against the metastatic spread of cancer cells [90,91,92]. Accordingly, there is a growing interest in immunotherapy strategies aimed at exploiting the anticancer potential of NK cells [74,91,92], as well as the very promising engineered NK cells [74,93] and, based on the results presented here, the efficacy of such approaches could be potentiated by the combined supplementation with CUR and RES. In addition, we observed a significant increase in CD68 expression in the monocyte/macrophages subset following RES and CUR + RES treatments which may suggest an increased monocyte/macrophage activation mediated by low-dose polyphenols [94].

## 4. Conclusions

**Figure 9:**
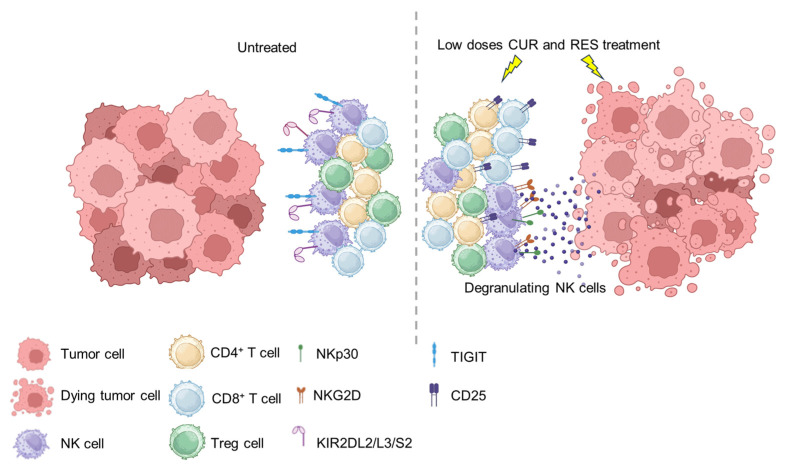
Proposed model illustrating the effects of CUR and RES combined treatment on tumor cells and PBMCs. The combination of bioavailable concentrations of CUR and RES reduced cancer cell survival without affecting PBMC viability, but instead increasing T cell activation (CD25^+^) and recognition of tumor by NK cells through the concomitant upregulation of the activating receptors (NKG2D and NKp30) and downregulation of the inhibitory and exhaustion receptors (KIR2DL2/L3/S2, KIR3DL1 and TIGIT). This figure was created using BioRender.com (accessed on 11 December 2023).

Overall, herein, we demonstrate that the combined use, at low doses, of CUR and RES can simultaneously reduce tumor cell growth [95,96] and shape immune responses (Figure 9).

For a prospective clinical use, the absence of toxicity of low-dose CUR and RES and the ease of application based on oral administration [97] make these polyphenols suitable tools that may be added to standard antitumor treatments including chemotherapy and radiotherapy [98], to biological agents such as immune-checkpoint inhibitors [99] and to adoptive immune therapies with T/CAR-T and NK/CAR-NK cells [100]. In this context, our findings may support the use of these polyphenols in combination with NK cell-based immunotherapies.

## 5. Materials and Methods

### 5.1. Tumor Cell Lines and PBMC

Human MM cell lines (H-Meso-1, MM-F1, MM-B1) were kindly provided by Prof. Antonio Procopio (Università Politecnica delle Marche, Ancona, Italy) and previously described [101,102]. Breast cancer (MCF7 and MDA-MB-468), head and neck carcinoma (SCC-15 tongue squamous carcinoma and A253 salivary gland carcinoma), prostate cancer (DU 145, PC-3), colon cancer (HCT 116), and erythro-leukemia (K562) cell lines were purchased from the American Type Culture Collection (ATCC, London, UK). Cells were cultured in DMEM high glucose medium with pyruvate (MM-F1, MM-B1, H-Meso-1, MCF-7, MDA-MB-468, DU 145, PC-3, HCT 116) or RPMI 1640 medium (A253, SCC-15 and K562) both supplemented with 10% FBS (Thermo Fisher Scientific, Whaltam, MA, USA), 2 mM L-glutamine, 100 I.U./mL penicillin and 50 µg/mL streptomycin (Euro Clone S.p.A., Milano, Italy).

Peripheral blood mononuclear cells (PBMCs) were obtained from buffy coats collected from anonymous healthy blood bank donors, in accordance with the Institutional Review Board of Bambino Gesù Children’s Hospital, IRCCS, Rome, Italy. PBMCs were isolated through density gradient centrifugation by Ficoll-Plaque Plus (Lympholyte Cedarlane, Burlington, NC, USA) and cryopreserved liquid nitrogen until further analysis.

### 5.2. Antibodies and Flow Cytometry

The following anti-human antibodies were used for flow cytometry: anti-CD3-AlexaFluor700 (UCHT1, BD Biosciences, San Jose, CA, USA), anti-CD14-AlexaFluor700 (HCD14, Biolegend, San Diego, CA, USA), anti-CD19-AlexaFluor700 (HIB19, Biolegend), anti-CD4-FITC (SK3, BD Biosciences), anti-CD4-PE (RPA-T4, BD Biosciences), anti-CD8-PerCP-eFluor 710 (SK1, Invitrogen-Thermo Fisher Scientific, Waltham, CA, USA), anti-CD25-PE (M-A251, BD Biosciences), anti-CD127-PE-Cy7 (HIL-7R-M21, BD Biosciences), anti-CD3-FITC (HIT3a, BD Biosciences), anti-CD68-PE (Y1/82A, Biolegend), anti-IFNγ-FITC or anti-IFNγ-APC (4S.B3, BD Biosciences), anti-IL-10-APC (JES3-9D7, Biolegend), anti-CD19-APC (HIB-19, BD Biosciences), anti-CD56-Pe-Cy7 (B159, BD Biosciences), anti-CD16 BV510 (3G8, BD Biosciences), anti-CD107a-FITC (H4A3, BD Biosciences), anti-NKG2D-PE-CF594 (1D11, BD Biosciences), anti-NKG2D-BV605 (1D11, BD Biosciences), anti-DNAM1-BV650 (11A8, BD Biosciences), anti-NKp46-PE-Cy7 (9E2, BD Biosciences), anti-NKp30-PE (Z25, BD Biosciences), anti-KIR3DL1-APC (DX9, R&D Systems, Minneapolis, MN, USA), anti-PD-1-BV421 (MIH4, BD Biosciences), anti-KIR2DL1/S1-PE-Cy5.5 (EB6B, Beckman Coulter, Brea, CA, USA), anti-NKG2A-FITC (REA110, BD Biosciences), anti-KIR2DL2/L3/S2-PE (GL-183, Beckman Coulter), and anti-TIGIT-APC (MBSA43, Invitrogen).

All the antibodies were used according to the manufacturers’ protocol. Prior to surface staining, PBMC and NK cells were pre-stained with Fixable Viability Dye eFluor™ 780 (Thermo Fisher Scientific). Before IFN-γ or IL-10 intracellular staining, cells were supplemented overnight with 1 µg/mL Brefeldin A (BFA, Merck-Italy-Sigma Aldrich, Milano, Italy) in order to enhance intracellular cytokine retention. Flow cytometry was performed by using FACSCanto (BD Biosciences) or Cytoflex (Beckman Coulter) and analyzed by FlowJo Software, version 10.0.8r1 (Treestar, Ashland, OR, USA), or CytExpert version 2.5 software. Before the assays, both CUR and RES were incubated individually or in combination with PBMCs for 96 h at 5 µM. DMSO, used as a solvent for both polyphenols, was used as a control.

### 5.3. Sulforhodamine B Assay

Tumor cell survival was evaluated by the sulforhodamine B (SRB) assay, as previously described [34]. Briefly, tumor cells were plated in flat bottomed 96-well plates at 2500 cells/well in 200 µL of medium. After 24 h, cells were incubated with 5 or 25 µM RES (cat no. R5010, purity ≥ 99%, Merck-Italy-Sigma Aldrich) and CUR (from *Curcuma longa*, cat. no. C1386, purity ≥ 65%, Merck-Italy-Sigma Aldrich) for 96 h. Cells were then fixed by adding 50 µL/well of 50% trichloroacetic acid (TCA, Merck-Italy-Sigma Aldrich) and incubated for 1 h at 4 °C. After 4 washings with distilled water, cells were dried and stained for 30 min with 100 µL of a 0.4% (*w*/*v*) SRB (Merck-Italy-Sigma Aldrich) solution in 1% acetic acid. The plate was washed 4 times with 1% acetic acid and left to dry. The dye was finally solubilized by adding 100 µL/well of 10 mM Tris pH 10. Cell density was then determined by spectrophotometric reading of the absorbance (O.D. values) 492 nm with a reference filter at 620 nm. The percentage survival of the cultures treated with RES and/or CUR was calculated by normalization of their O.D. values to those of the control cultures treated with DMSO [34].

### 5.4. PBMC Proliferation and Cell Death Assays

PBMC proliferation was evaluated through flow cytometric measurement of carboxyfluoresceinsuccimide ester (CFSE) dye dilution. PBMCs were thawed, counted, and stained with 0.5 µg/mL CFSE (CellTrace Cell Proliferation Kit, Invitrogen-Thermo Fisher Scientific) for 15 min at 37 °C. At the end of the incubation, the cells were washed in complete RPMI medium, plated in 96-well U-bottom plates at a concentration of 300,000 cells/well and treated with 5 µM CUR and/or RES for 96 h. DMSO was used as a control.

The percentage of necrosis and apoptosis of PBMCs treated with CUR and/or RES for 96 h was evaluated by using a PE-conjugated Annexin V/7AAD apoptosis detection Kit (Biolegend) and flow cytometric analysis.

### 5.5. Reactive Oxygen Species Detection Assay

The production of reactive oxygen species (ROS) was evaluated in PBMCs plated in 96-well U-bottom plates at a concentration of 300,000 cells/well. Cells were pre-treated with 5 µM CUR and/or RES, or DMSO, for 96 h and then incubated with phorbol-12-myristate-6-acetate (PMA, 50 ng/mL, Merck-Italy-Sigma Aldrich) for 90 min. During the last 30 min of incubation with PMA, the fluorogenic probe dichlorodihydrofluorescein diacetate (DCFDA, 20 µM, Merck-Italy-Sigma Aldrich) was added to cultures and then green emission was detected by flow cytometry.

### 5.6. NK Cell Degranulation Assay

Degranulation assay was performed by co-culturing PBMCs, untreated or pre-treated with 5 µM CUR and/or RES for 48 h, with K652 target cells at a 1:1 ratio for 3 h in complete medium in the presence of anti-CD107a at a 1:100 dilution. During the last 2 h of co-culture, GolgiStop (BD Biosciences), used at a 1:500 dilution, was added. Cells were then washed, centrifuged, and stained with anti-CD56, anti-CD16, anti-CD3, anti-CD14 and anti-CD19 to evaluate CD107a expression in the CD56^+^CD16^+^CD3^−^CD14^−^CD19^−^ subset by flow cytometry.

### 5.7. Statistical Analysis

Data distribution of cell growth and apoptosis assays was preliminarily verified using the Kolmogorov–Smirnov test, and the datasets were analyzed by one-way analysis of variance (ANOVA) followed by the Newman–Keuls test. For all other data, statistical significance was evaluated with the unpaired or paired two-tailed Student’s *t*-test. Normalized values were analyzed for correlation by the regression analysis using GraphPad Prism version 5.0 software. Values with *p* ≤ 0.05 were considered to be statistically significant.

## Data Availability

Data is contained within the article or Supplementary Material.

## Supplementary Materials

The following supporting information can be downloaded at: https://www.mdpi.com/article/10.3390/ijms25010232/s1.
