# Supplementary material for: The Combination of Bioavailable Concentrations of Curcumin and Resveratrol Shapes Immune Responses While Retaining the Ability to Reduce Cancer Cell Survival
Source: Int J Mol Sci. 2023 Dec 23;25(1):232. doi: 10.3390/ijms25010232 (PMC10779126; doi:10.3390/ijms25010232)
Supplement: Supplementary file 1 [file ijms-25-00232-s001.zip › ijms-2711115-supplementary.pdf]

Supplementary Materials
ijms-2711115

The Combination of Bioavailable Concentrations of Curcumin and Resveratrol Shapes Immune Responses While Retaining the Ability to Reduce Cancer Cell Survival

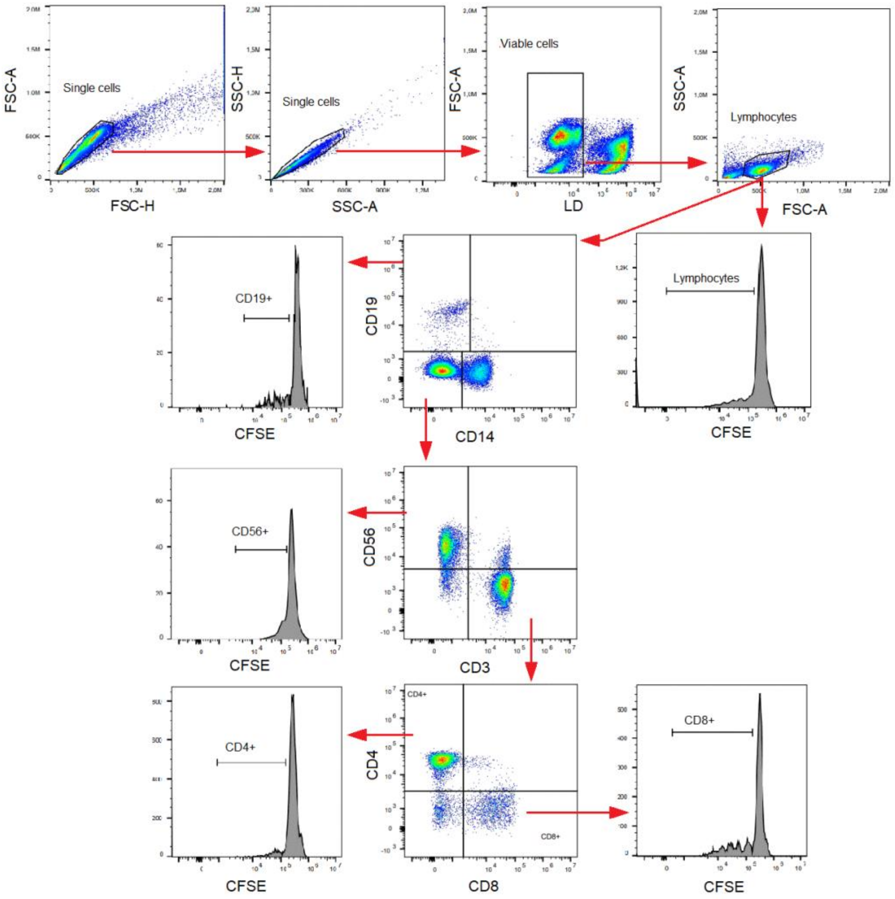

Figure S1: Gating strategy used to analyse cell subsets by flow cytometry. PBMCs from healthy donors, untreated or treated with 5 μM CUR and/or RES for 96 hours, were checked for the expression of B, NK, and T cell specific markers to perform immunophenotyping analysis. After gating single cells (forward scatter A versus forward scatter-H and side scatter-A versus side scatter-H), and viable cells (negative for Live/Dead staining), B cells were identified by gating on $CD14^-CD19^+$, NK cells were identified by gating on $CD14^-CD19^-CD3^-CD56^+$, helper or cytotoxic T cells were identified by gating on $CD14^-CD19^-CD3^+CD56^-CD4^+$ or $CD14^-CD19^-CD3^+CD56^-CD8^+$ respectively. CFSE expression level was then evaluated on each subset. A representative staining of untreated PBMCs from a healthy donor is shown.

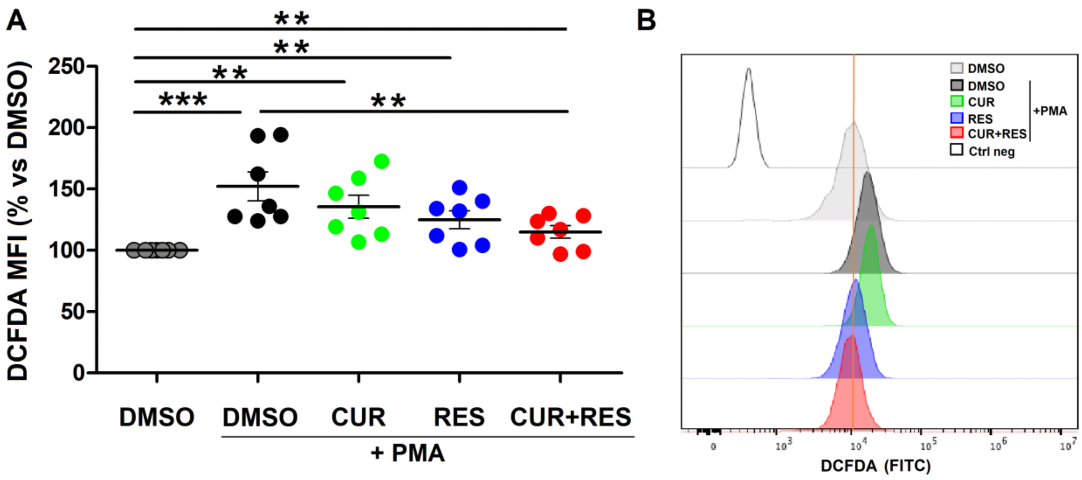

Figure S2: Effect of CUR and RES on reactive oxygen species (ROS) generation in PBMCs. A) ROS were induced by treatment with PMA (50 ng/ml, 90 minutes) after 96 hours of PBMCs treatment with DMSO, CUR, and/or RES (5 µM). ROS production was assessed by flow cytometry through the green fluorescence generated by DCFDA oxidation. The results are presented as the mean ± SD of the frequency in PBMCs from 7 healthy donors. Dots correspond to the mean of fluorescence (MFI) normalized to that of control cultures treated with DMSO and not stimulated with PMA. Statistical significance of the effects obtained with CUR and RES, alone or in combination, was calculated with two-tailed unpaired Student's t test (**p≤0.01; *** p≤0.001). B) Representative histograms of DCFDA green emission upon different treatments.

Supplementary Figure 3

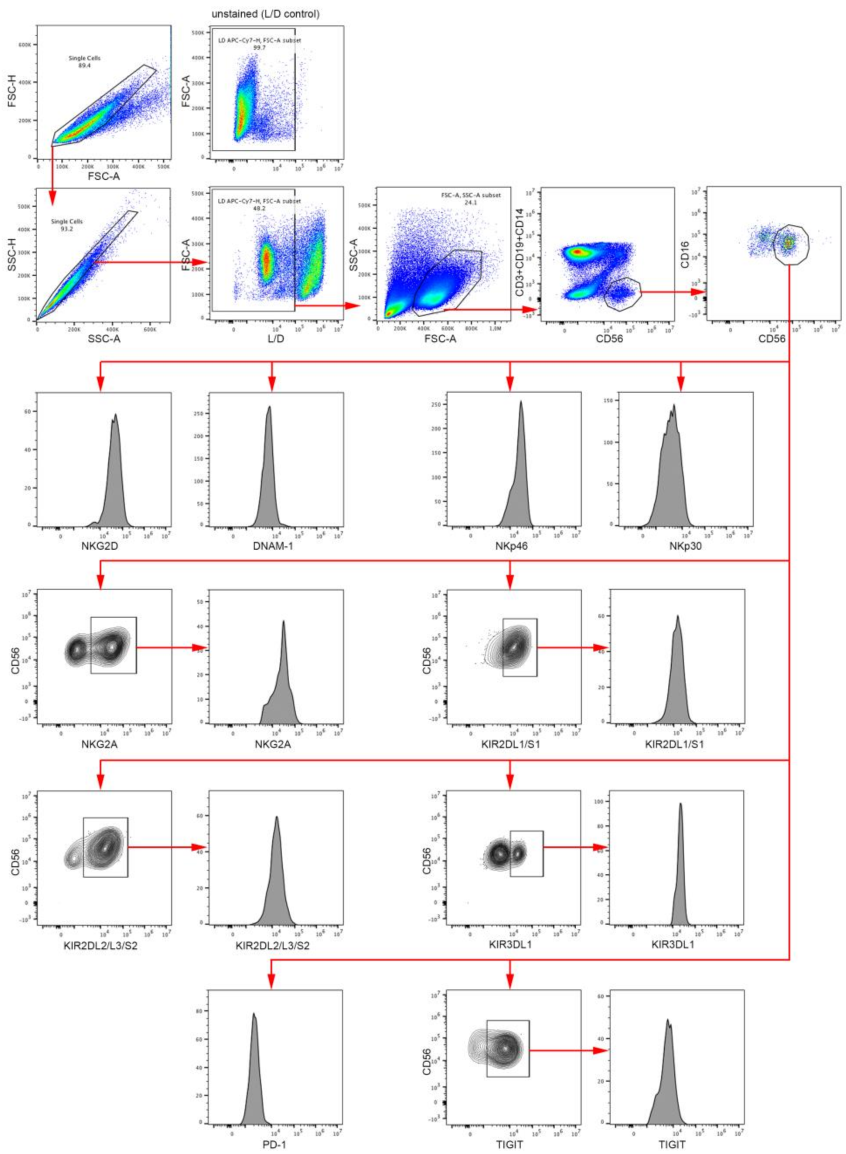

Figure S3: Gating strategy used to analyze NK cell subsets by flow cytometry. PBMCs from healthy donors, untreated or treated with 5 μM CUR and/or RES for 48 hours, were checked for the expression of NK cell specific markers to perform both degranulation assay and immunophenotyping analysis. After gating single cells (forward scatter A versus forward scatter-H and side scatter-A versus side scatter-H), and viable cells (negative for Live/Dead staining), NK cells were identified by gating on $CD14^{-}CD19^{-}CD3^{-}$ subsets to evaluate in $CD56^{+}CD16^{+}$ subset the expression of surface markers, as indicated. A representative staining of untreated PBMCs from a healthy donor is shown.
